# The polypill approach – An innovative strategy to improve cardiovascular health in Europe

**DOI:** 10.1186/s40360-016-0102-9

**Published:** 2017-02-07

**Authors:** Valentín Fuster, Francesc Gambús, Aldo Patriciello, Margaretha Hamrin, Diederick E. Grobbee

**Affiliations:** 1 Fundación Centro Nacional de Investigaciones Cardiovasculares Carlos III, Madrid, Spain; 2 Icahn School of Medicine at Mount Sinai, New York, USA; 3 Group of the European People’s Party (Christian Democrats), European Parliament, Rue Wiertz, Altiero Spinelli 11E261, 1047 Brussels, Belgium; 4 Group of the European People’s Party (Christian Democrats), European Parliament, Rue Wiertz, Altiero Spinelli 10E209, 1047 Brussels, Belgium; 5 Familial Hypercholesterolemia Foundation Norway, FH Norge, PB 9569, Youngstorget, 0028 Oslo, Norway; 6 Julius Global Health, Julius Center for Health Sciences and Primary Care, University Medical Center Utrecht, 3508 GA Utrecht, The Netherlands

## Abstract

**Background:**

Cardiovascular disease (CVD) is a major cause of disability and premature death. Despite European guidelines advocating the use of medical therapies in CVD, many patients still do not achieve the guideline-recommended treatment, which highlights the need for change and innovations in this field. This requirement has been widely recognised by the national ministries of health, several European cardiology societies, and the European Parliament, who support the initiation of strategies to improve and promote cardiovascular health.

**Discussion:**

One of the key risk factors to recurrent cardiovascular events is the lack of adherence to medication and this has been added to the agenda of the European Commission. With the intention to improve treatment adherence in CVD, polypills have been investigated and numerous studies demonstrate that they significantly improve medication adherence, which contributes to the improvement of health outcomes. In Europe, the first cardiovascular polypill, developed by a public-private partnership (CNIC-Ferrer), recently became available for general prescription as a therapy for CVD prevention. This polypill significantly improves adherence, preventing fatal and non-fatal cardiovascular events, and appears to be a cost-effective strategy to improve sustainability of the health care systems in CVD.

**Conclusions:**

Given the importance of urgent and simple solutions to restraining the pandemic nature of CVD, the polypill approach should therefore be considered by physicians and public health systems as an available and innovative option to improve cardiovascular health.

## Background

Cardiovascular disease (CVD) is a major cause of disability and premature death worldwide. Despite European and American guidelines advocating the use of medical therapies in CVD, many patients still do not achieve the guideline-recommended treatment, due to several reasons including poor or non-adherence to the prescribed therapy or high medication burden. As such, there is a clear need for change and innovation in this field.

This need has been widely recognised in political, scientific and patient communities in their support of the initiation of strategies to improve and promote cardiovascular (CV) health.

One of the key risk factors to recurrent CV events is the lack of adherence to medication and this has been added to the agenda of the European Commission. With the intention to improve treatment adherence and strengthen comprehensive CVD prevention plans, several approaches and interventions have been analysed, such as the use of Dual Antiplatelet Therapy (DAPT), as well as different tactics to modify behavioural risk factors. There have, however, only been few advances in the field of drug treatment aimed at enhancing treatment effectiveness. In particular, polypills have been investigated in the CVD field and numerous studies demonstrate that they significantly improve medication adherence, which contributes to the improvement of health outcomes.

This article analyses the issue of poor and non-adherence to medication as a risk factor for CVD prevention and focuses on the polypill therapy as an effective approach to help reduce the number of recurring CV events in Europe.

## Discussion

### Epidemiology and burden of CVD in Europe

It has been widely demonstrated that CVD is a major cause of disability and premature death worldwide [1]. An estimated 17.5 million people died in 2012 as a consequence of CVD [2], and it is expected that this figure will increase by 2030, reaching 23.3 million deaths directly related to CV events [1].

Looking at a regional level, CVD is the leading contributor to mortality in the 53 countries of the World Health Organization (WHO) Europe Region, causing almost 4.1 million deaths each year, which means 46 % of all deaths in Europe. In the European Union (EU) alone, CVD causes more than 1.9 million deaths annually and the geographical distribution of this figure across Europe reflects particularly higher rates of deaths in the northern countries over the southern nations. In all countries, death rates for coronary heart disease (CHD) are higher in males than females [3].

The global burden of CVD is led by CHD and stroke, which have been identified as the first and third lead diseases for disability-adjusted life-years, as a sum of years of life lost due to premature death and years of life lived with disability, worldwide [4]. For Europeans, in addition to being the lead cause of mortality, CVD also makes a substantial contribution to morbidity rates. Overall, CVD is estimated to cost the European economy almost EUR 196 billion a year. Of the total spending, around 54 % is directly associated to health care costs, 24 % to productivity losses and 22 % is a consequence of the informal care of people with CVD [5].

### The importance of secondary prevention in CVD

Some studies showed that the progresses made in securing the stabilisation of patients after a CV event, such as myocardial infarction (MI), have largely contributed to the prevalence of CHD as a chronic disease. Consequently, recurrent CVD events are common among people who have previously been diagnosed with an MI, with rates found as close to 50 % for any CVD event [6, 7] or subsequent revascularisation [8] during the first year after suffering an MI. It has also been demonstrated that up to 75 % of patients have a recurrent event within 3 years after suffering a MI [7, 9]. In addition, patients with a history of acute myocardial infarction (AMI) are at least five to six times more likely to die of CV causes within a year [10].

It is therefore essential to understand and to consider the implications for the long-term prognosis of the condition, both for survival and the risk of recurrence [11]. In this regard, guidelines have suggested behavioural risk factors as critical for the progression of CVD in patients, such as smoking, unbalanced diet, lack of physical activity and harmful use of alcohol [12]. In addition, medical risk factors, such as no revascularization procedures, the presence of comorbidities, as well as the lack of adherence to prescribed medication, have also been shown to be associated with recurrent CV events [13].

Although modifying patients’ lifestyle and behaviours could partly reduce the recurrence of CV events, [12], a large body of evidence supports the use of medical therapies as the most effective approach to secondary prevention of CVD [14, 15]. The combined use of aspirin, angiotensin-converting-enzyme (ACE) inhibitors and lipid-lowering therapies has been proven [16, 17] to be highly effective in lowering the risk of secondary CV events. Indeed, it has been estimated that about two-thirds to three-quarters of future vascular events could be prevented when using these drugs together [15], and it also leads to a large reduction of CVD-related deaths [14]. Given this evidence, decreasing the recurrence of CV events has become a global priority [18], and, accordingly, European guidelines have openly and strongly advocated for the use of medical therapies in the prevention of secondary CV events and in particular for the use of polypills to increase adherence [19].

However, while guidance from experts is clear, recent studies have demonstrated a suboptimal use of medicines targeted for the prevention of recurrent CV events, showing that only 43 % of patients with acute coronary syndrome (ACS) are actually prescribed with optimal treatment for secondary prevention [20, 21]. As a consequence, experts have made a call to action, which highlights the need to improve clinical prevention particularly in primary care, where it may be beneficial to enhance the quality of the relationship between healthcare providers and patients to ensure that they benefit from fully available knowledge [10].

In addition, the latest EUROASPIRE study [22], a cross-sectional study undertaken across 78 centres in 24 European countries, demonstrated that a large majority of coronary patients does not follow the recommendations set by guidelines on modifying their behavioural patterns towards a balanced and healthy lifestyle. Therefore, risk factor control and therapeutic targets are not achieved as required to manage the prevention of recurrent CV events properly.

Adherence to medication has been widely identified as a risk factor to the recurrence of CVD. Good adherence is associated with positive health outcomes and poor adherence to treatment actually increases the likelihood of suffering a recurrent CV event [23, 24]. A recent study further showed that low-risk, intermediate risk and high-risk patients with AMI in the year following discharge, present poor adherence rates to prescribed therapies: 61.5, 57.9 and 45.9 % respectively [25]. Supporting this finding, a previous meta-analysis also demonstrated that compliance with the medication was met only in 66 % of CVD cases [26].

A direct link between adherence to treatment and health outcomes has been repeatedly reported and poor compliance was documented to be associated with considerable worsening of the condition [27], rehospitalisation, morbidity and mortality [28]. In order to reduce low adherence rates, many experts have studied the link between compliance with medication and pill burden as a potential key to modifying treatment outcomes through adapting patient’s behavioural patterns. As a result, it has been shown that the prescribed number of doses per day is inversely correlated to adherence, and simpler and less frequent dosing regimens usually drive better compliance rates [29]. This data therefore supports the view that optimization of treatment regimens and increased compliance can help to prevent the recurrence of CV events [22].

Given the evidence, it is clear that medication adherence in CVD prevention remains a challenge for patients and healthcare providers. Lack of compliance is not only associated with a lower quality of life and poor health outcomes, but also has socio-economic impact and generates elevated costs to healthcare systems. In the EU, the number of deaths associated with miss-dose and non-adherence to prescribed medication in general is estimated to be approximately 194,500 per year and non-adherence is estimated to cost the EU EUR 125bn annually [30].

### The need for change and innovation in CVD prevention

The need for new and more effective delivery of evidence based preventive treatments to patients is increasingly recognised by Governments of Member States of the EU, the European Society of Cardiology (ESC), National Cardiac Societies, the European Heart Network (EHN), National Heart Foundations and the Union of European Medical Specialists Union (Européenne des Médecins Spécialistes, UEMS) Cardiology section. The importance of strengthening of comprehensive CVD prevention plans and the certainty that effective measures, policies, and interventions would be in place in all European countries, was adopted in 2005 in the Luxembourg Declaration [31]. In 2007, the European Parliament also adopted by a large majority a ‘Resolution on Action to Tackle Cardiovascular Disease’, calling on the Commission and Member States to adopt or review national public health strategies to include the promotion of strategies on CV health amongst others [32]. More recently, the MEP Heart Group, in collaboration with the WHO and supported by the ESC and the EHN, launched the *Pledge for Cardiovascular Health*, encouraging members of the European Parliament (MEPs) to show their support for CV health and committing them to consider the impact of CV health when voting on EU legislation and to support national strategies to promote CV health [33].

### Recent innovations in the prevention of CV events

In line with these calls for action, clear advances in the field were made and some of the key progresses in CVD prevention in 2015 were summarised [34]. These include the introduction of a comprehensive personalised preventive strategy to reduce modifiable risk factors, including lifestyle and dietary habits. Among the main outcomes observed through the control and close follow-up of behavioural factors in patients at high risk of CVD, a significant reduction in events was demonstrated over a 5-year period [35].

Advances in the treatment of CVDs from previous years that target non-modifiable risk factors include the use of Dual Antiplatelet Therapy (DAPT), which is usually prescribed after a heart attack or stent placement to keep the vessels open and to prevent future heart attacks [36]. The administration of DAPT for 12 months in patients with ACS, was shown to improve the outcomes when compared to administrating aspirin alone for the prevention of recurrent events [37].

Despite these calls for action, there have only been a few clear advances in drug treatment in the field in recent years.

The polypill approach, which combines several medicines that simultaneously control several risk factors or disease mechanisms in a single pill, is one of those strategies that has, in recent years, progressed in the CVD field. The concept was introduced by Wald and Law in 2003, who described a fixed-dose combination strategy, containing six components, and claimed that the administration of this polypill to each individual over 55 years old would reduce the incidence of CVD by more than 80 % [38]. Since then, several polypill concepts have been proposed, including the ‘vaccination approach’, which refers to the use described by Wald and Law, as well as the use in primary and secondary CVD prevention [39]. As with most treatments, the benefits, as well as the drawbacks of the polypill, have been widely discussed [39]. Some argued that the originally proposed estimated risk reduction potential of the polypill could be too optimistic and that many patients would remain undertreated. Concern about potential adverse effects related to some of the polypill’s monocomponents also exist, and it has been argued that side effects from one of the components could lead to discontinuation of treatment. This could then result in the loss of all the benefits of the other components included in the polypill [39]. Nonetheless, despite these concerns, the potential of the polypill to improve the management of CVD risk factors has been recognised by several expert panels, including the WHO and the Combination Pharmacotherapy and Public Health Research Working Group, who consider research in this area an important breakthrough [39].

### The polypill approach in CVD prevention

Several drug classes (antiplatelet agents, beta-blockers, renin-angiotensin system blockers and statins) exist, and have been shown in multiple large cardiovascular outcome trials to reduce the risk of events when used in the setting of CVD prevention [19]. However, implementing preventative treatment in routine care has been less successful and several barriers exist; notably, insufficient prescription of therapies to patients and poor adherence to medicines by patients.

The polypill approach has been advocated to help overcome some of these barriers to CVD prevention [19]. To date, three polypills have been investigated for CVD prevention. The CNIC-Ferrer polypill is, however, the only one for which a marketing authorisation has been granted in the EU, in other European countries and in Latin-America so far [40].

The effect of polypills on treatment adherence has been studied in detail in recent years. As such, results from the UMPIRE, IMPACT, Kanyini GAP, and FOCUS trials showed that the polypill significantly increases adherence to treatment when compared to administering either the individual drugs separately [41] or when compared to usual care [42–44].

In more detail, the randomised, open-label UMPIRE trial among people with established CVD or at risk of CVD demonstrated that the use of a fixed-dose combination (FDC) therapy significantly improved adherence to treatment at 15 months, as well as leading to statistically significant improvements in systolic blood pressure (SBP) and low-density lipoprotein (LDL) cholesterol [42]. In line with this, the open label randomised control trial IMPACT (IMProving Adherence using Combination Therapy) assessed the effects of FDC treatment on adherence and risk factor control, compared with usual care of patients at high risk of CVD in primary care. The authors showed that adherence to recommended medications was greater among FDC than usual care participants at 12 months (81 % versus 46 %). A post-trial survey of this study further revealed a high acceptability of the FDC therapy for general practitioners and patients [43]. Similarly, another randomised clinical trial conducted in 623 patients with established CVD or at high risk of CVD – the Kanyini GAP trial – compared the polypill to usual care and showed that patients treated with the polypill were more adherent to their treatment than patients at usual care after a follow up at 18 months [44]. The three trials described above were part of the Single Pill to Avert Cardiovascular Event (SPACE) collaboration, which compared polypill-based care with usual care. A prospective, individual participant data meta-analysis of these three controlled trials was published late 2015, showing that the polypill therapy significantly improved adherence, SBP and LDL-cholesterol in high risk patients when compared with usual care, especially among those patients who were under-treated at baseline [45].

Moreover, the cross-sectional FOCUS study (Fixed-Dose Combination Drug for Secondary Cardiovascular Prevention) assessed the effect on adherence of treatment with the polypill in comparison to treatment with its separate monocomponents in 695 adult patients following a MI. After 9-months of follow-up, the percentage of adherent patients in the polypill group was significantly increased by approximately 22% when compared to the group receiving the separate components of the polypill [41].

A more general meta-analysis assessing patients with acute and chronic diseases also examined the impact of reduced frequencies of oral therapies from multiple- to once-daily dosing schedule on adherence. It suggested that across acute and chronic disease states, adherence to therapies might be improved by reducing dosage frequency. The study further suggested that this increase in adherence may result in subsequently lower healthcare costs [46]. In line with this, several other articles also found that a simpler and less frequent dosing regimen results in improved compliance across a variety of therapeutic classes [29], and adherence rates decrease when dosing frequency increases [47].

By reducing the complexity of the medication regimen, the polypill can also help overcome some of the additional risks and problems associated with polypharmacy. Polypharmacy after a CV event is common and may pose risks, such as adverse drug interactions, accidental overdosing and medication non-adherence, that originate from the difference in medication schedule and dosage [48]. Especially in elderly patients, polypharmacy may pose a problem due to the patient’s strong belief in their medication and self-medication, which can lead to adverse drug interactions and other mistakes in their drug intake [49, 50]. By reducing the pill burden and by improving medication compliance, the polypill has thus been suggested as a way to help overcome some of the issues related to polypharmacy [51, 52], as well as translating into better CV outcomes.

Interestingly, numerous studies have demonstrated that patients also consider polypills as more convenient and prefer having to take one single medicine instead of several individual medicines. Indeed, a recent study assessing the influence of polypill-based treatment attributes and patient characteristics on preferences for CVD preventive treatment showed that treatment preference decreased with tablet number. The discrete choice experiment further demonstrated that patients value and are willing to pay a premium for each tablet reduction [53].

The polypill provides physicians with an improved tool to meet the progressively more stringent secondary prevention guidelines from the ESC and other medical societies. This is significant as inadequate prescription of medicines remains a substantial gap in the coverage of secondary interventions for the prevention of CVD, as demonstrated by the findings from the WHO study on Prevention of REcurrences of Myocardial Infarction and StrokE (WHO-PREMISE) [10], the Antiplatelet Treatment Observational Registry (APTOR) [20], SURF [21], and EUROASPIRE IV study [22].

Despite the impressive number of potential benefits of the polypill strategy in CVD prevention, its use is relatively novel. One concern of polypill sceptics is that patients will regard the polypill as an excuse to potentially replace efforts to promote healthy lifestyles [54]. Rather than replacing them, lifestyle modifications should be initiated simultaneously with drug therapy as the benefits associated are additive and complement each other in the prevention of CVDs. Based on the WHO’s recommendations, the polypill approach could be a ‘best buy’ as a primary healthcare approach for CVD prevention [12].

### The CNIC-Ferrer polypill project

Polypill is now a reality because the first of its kind has been released to the market in Europe. The CNIC-Ferrer polypill project is the European polypill developed as a therapy for secondary CVD prevention. It was inspired by the needs of most patients with a previous heart disease, guideline recommendations outlined above, and negative health outcomes originating from lack of adherence. It was developed through a public-private strategic partnership between the Centro Nacional de Investigaciones Cardiovasculares (CNIC), Spain, and Ferrer, Spain. The research was supported by European funding from the 7^th^ European Framework Programme and *Horizon 2020* to help overcome the unmet clinical need in CVD prevention [55].

To date, the CNIC-Ferrer polypill strategy is the first and only polypill approved in 15 European countries [56], already commercialised and reimbursed in several ones, commercialised in various Latin American countries as well as under registration in some Middle-East and North African (MENA) and Asia-Pacific (APAC) countries. The polypill is expected to simplify the therapy by reducing the patient’s pill burden, which may improve adherence to secondary prevention medications and decrease further CV events in the long term.

This polypill consists of an angiotensin-converting enzyme (ACE) inhibitor (ramipril), a statin (atorvastatin) and an aspirin, at dosages established to achieve the best balance safety/benefit that has been proven to prevent CVD events. Moreover, the use of the polypill components in patients who have suffered a CV event is commended by ESC [19], American Heart Association (AHA) [57], and WHO guidelines [18]. In addition, the CNIC-Ferrer polypill has proven bioequivalence compared with each of its original mono-components and holds a patent for its innovative galenic formulation [56].

By combining three medicines recommended for the secondary prevention of CV events in a once-daily capsule, the polypill was shown to significantly increase adherence in patients following a CV event when compared to administering the three drugs separately [41]. This increase in adherence has been suggested to lead to a reduction of recurrent CV events and its associated costs, which originate from rehospitalisation, long hospital stays and revascularization procedures [12, 53, 58]. Indeed, recently published studies demonstrated that the use of a polypill appeared to be a cost-effective strategy to prevent fatal and non-fatal CV events in the UK [59], Spain [60], and Mexico [61].

### A pan-European perspective

One of the priorities of the European Commission is to contribute to the improvement of adherence to medical plans and medication [62]. Furthermore, in the broader European CVD environment, efforts continue to deliver patient access to the most compelling, evidence-based preventative therapies. The European Commission’s (EC) *Horizon 2020* framework programme, which is making EUR 80 billion of public funding available to researchers between 2014 and 2020 [63] alongside private sector investment, is actively investing in research and innovation for long-term chronic diseases, including CVD [58, 64], and has been highlighted as a critically-important vehicle to innovate for improved patient outcomes. Of note, amongst other innovations in chronic diseases, the potential role of polypill-based strategies in CVD prevention is also being investigated within the *Horizon 2020* framework [55].

There is a pan-European fiscal challenge facing the management of non-communicable diseases (NCDs), such as CVD. Heart disease, stroke and diabetes cause significant loss of national income each year in the world’s most populous nations. Economic analysis suggests that each 10 % rise in NCDs is associated with 0.5 % lower rates of annual economic growth [12].

### Public-private partnerships to unlock improved CVD outcomes

The International Monetary Fund (IMF) has highlighted public-private partnership as a very relevant way to improve R&D and innovation [65]. These types of partnerships have already proven successful in several therapeutic areas. Challenging public-health diseases such as malaria and sleeping sickness has demonstrated not only the importance, but how crucial is the role of a such public health-oriented R&D in order to ensure timely and effective delivery of new drugs to face very important public-health diseases [66, 67].

The CNIC-Ferrer polypill project discussed in this paper is a recent example of a successful collaboration between publicly-funded health bodies and the industry sector to deliver improved therapeutic strategies and solutions in chronic disease areas. CNIC, supported by the Ministry of Economy of the Spanish Government, aims to expedite CV research in Spain and to ensure funding is made available to conduct biomedical research with a focus on prevention rather than treatment, where possible. Its objectives are aligned with the EC *Horizon 2020* vision of reducing ‘red tape’ in a bid to ensure Europe produces world-class science, removes barriers to innovation and makes it easier for the public and private sectors to work together. The author group of this paper believe the CNIC-Ferrer partnership provides an appealing and innovative model for therapeutic discovery and development on the continent.

### A call to action

Combination therapy has become the standard for treating several relevant major communicable diseases such as HIV/AIDS, TB and malaria, and has recognised benefits in slowing resistance, improving clinical outcomes and facilitating logistics [68]. Other non-communicable diseases such as hypertension [69] and diabetes [70], have also been proven to benefit from combination therapy.

The European Medicines Agency (EMA) recognises that fixed-dose combinations of medicinal products have been increasingly used due to the benefit of the combined effects of active substances given together and has issued a guideline on the clinical development needed for ‘fixed dose combination’ medicinal products depending on the intended indication [71].

CVD prevention remains one of the key medical challenges affecting European populations, and effective, simple solutions have a key role to play in restraining the pandemic nature of the disease [54]. The polypill approach to CV prevention, in particular, has shown significant potential in the absence of a plethora of alternative strategies which do not rely on a multi-drug approach. It has already shown an adherence benefit, and can be sustained across multiple geographies. Additional research is ongoing; however such is the scale of the public health challenge in CVD that health professionals should consider the polypill approach among the available options (in harmony with lifestyle modifications) in the context of individualised patient need. The authors also urge European public health systems to consider endorsing this valuable innovation for their patients.

Moreover, in view of the public-health interest to assure adherence to treatment in relevant communicable and non-communicable diseases, additional programmes on adherence and more research should be undertaken and funding provided to gather evidence, in order to healthcare professional to tackle CVD burden by adopting innovative healthcare interventions including the polypill strategy. Last but not least, European patient associations should envisage educational training to promote patients’ awareness of cardiovascular risk and disease while engaging them in the adherence to healthy lifestyle habits and pharmacological treatment with the final objective to control risk factors and empower them.

The authors’ call to action is therefore directed to payers, patient associations, industry, research funders, regulators, and healthcare professionals, to ensure that polypills are developed, made available and accessible to patients in diseases than benefit from improving the adherence to their treatments.

## Conclusions

Following an analysis of the current context of CVD in Europe, a need for innovative and effective interventions in the prevention of secondary CVD was highlighted.

In order to cope with poor and non-adherence to treatment, which has been widely identified as a key risk factor in the prevention of CVD, the polypill approach has been demonstrated to be an effective innovation to improve CVD outcomes. Several studies have shown that a simpler and less frequent dosing regimen results in improved compliance to medication across a variety of therapeutic classes and that adherence rates decrease when dosing frequencies increase. In line with this, the polypill, which reduces the complexity of the medication regimen, was demonstrated to help increase medication adherence in CVD patients, improving health outcomes.

In light of the epidemic scale of CVD, the first CV polypill was recently made available by European decision-makers as a therapy for CVD prevention in Europe. This polypill was shown to significantly improve adherence and reduce the number of CV events. It also appeared to be a cost-effective strategy to prevent fatal and non-fatal CV events.

It is clear that CVD prevention remains to be one of the key medical challenges in Europe, and effective, simple solutions are needed to restrain the pandemic nature of the disease. The polypill approach has a significant potential in the context of CVD prevention and should be considered an available, valuable innovation by healthcare professionals, patients and public health systems in order to improve CV health.

## Abbreviations

ACE: Angiotensin-converting-enzyme
ACS: Acute coronary syndrome
AHA: American Heart Association
AMI: Acute myocardial infarction
APAC: Asia-Pacific region
CHD: Coronary heart disease
CNIC: National Centre for Cardiovascular Research (from the Spanish, Centro Nacional de Investigaciones Cardiovasculares)
CV: Cardiovascular
CVD: Cardiovascular disease
DAPT: Dual antiplatelet therapy
EC: European Commission
EHN: European Heart Network
ESC: European Society of Cardiology
EU: European Union
FDC: Fixed-dose combination
IMF: International Monetary Fund
LDL: Low-density lipoprotein
MENA: Middle East and North African region
MEP: Member of the European Parliament
MI: Myocardial infarction
NCD: Non-communicable disease
SBP: Systolic blood pressure
UEMS: Union of European Medical Specialists (from the French, Union Européenne des Médecins Spécialistes)
WHO: World Health Organization.
